# Gut microbiome alterations in preclinical Alzheimer’s disease

**DOI:** 10.1371/journal.pone.0278276

**Published:** 2022-11-29

**Authors:** Joon Hyung Jung, Gihyeon Kim, Min Soo Byun, Jun Ho Lee, Dahyun Yi, Hansoo Park, Dong Young Lee, for the KBASE Research Group

**Affiliations:** 1 Department of Psychiatry, Seoul National University College of Medicine, Seoul, Republic of Korea; 2 Department of Neuropsychiatry, Seoul National University Hospital, Seoul, Republic of Korea; 3 Department of Biomedical Science and Engineering, Gwangju Institute of Science and Technology (GIST), Gwangju, Republic of Korea; 4 Institute of Human Behavioral Medicine, Medical Research Center Seoul National University, Seoul, Republic of Korea; 5 Genome and Company, Seongnam, Republic of Korea; Indiana University Purdue University at Indianapolis, UNITED STATES

## Abstract

**Background:**

Although some human studies have reported gut microbiome changes in individuals with Alzheimer’s disease (AD) dementia or mild cognitive impairment (MCI), gut microbiome alterations in preclinical AD, i.e., cerebral amyloidosis without cognitive impairment, is largely unknown.

**Objective:**

We aimed to identify gut microbial alterations associated with preclinical AD by comparing cognitively normal (CN) older adults with cerebral Aβ deposition (Aβ+ CN) and those without cerebral Aβ deposition (Aβ− CN).

**Methods:**

Seventy-eight CN older participants (18 Aβ+ CN and 60 Aβ− CN) were included, and all participants underwent clinical assessment and Pittsburg compound B–positron emission tomography. The V3–V4 region of the 16S rRNA gene of genomic DNA extracted from feces was amplified and sequenced to establish the microbial community.

**Results:**

Generalized linear model analysis revealed that the genera *Megamonas* (B = 3.399, q<0.001), *Serratia* (B = 3.044, q = 0.005), *Leptotrichia* (B = 5.862, q = 0.024) and *Clostridium* (family *Clostridiaceae*) (B = 0.788, q = 0.034) were more abundant in the Aβ+ CN group than the Aβ− CN group. In contrast, genera *CF231* (B = −3.237, q< 0.001), *Victivallis* (B = −3.447, q = 0.004) *Enterococcus* (B = −2.044, q = 0.042), *Mitsuokella* (B = −2.119, q = 0.042) and *Clostridium* (family *Erysipelotrichaceae*) (B = −2.222, q = 0.043) were decreased in Aβ+ CN compared to Aβ− CN. Notably, the classification model including the differently abundant genera could effectively distinguish Aβ+ CN from Aβ− CN (AUC = 0.823).

**Conclusion:**

Our findings suggest that specific alterations of gut bacterial taxa are related to preclinical AD, which means these changes may precede cognitive decline. Therefore, examining changes in the microbiome may be helpful in preclinical AD screening.

## Introduction

A growing body of evidence indicates that alterations in the gut microbiome are associated with various brain diseases via the so-called brain–gut–microbiota axis [1], as well as with other systemic diseases such as obesity, Type II diabetes mellitus (DM) [2], and systemic lupus erythematosus [3].

Particularly with regard to Alzheimer’s disease (AD), recent animal studies have strongly suggested a relationship between gut microbial alteration and the development of the disease. A study demonstrated that the gut microbiome was altered in APP/PS1 mice, and such alteration was related to increased cerebral beta-amyloid (Aβ) burden [4, 5]. Additionally, cerebral Aβ deposition was significantly reduced in germ-free APP/PS1 mice, whereas recolonization of these mice increased cerebral Aβ levels [4]. Moreover, a recent study revealed that transplantation of a healthy gut microbiome reduced Aβ deposition in ADLP^APT^ mice, recently developed AD-like pathology transgenic mice [6].

Several human studies have also demonstated that gut microbiome composition differed in individuals with clinically defined Alzheimer’s disease (AD) dementia or mild cognitive impairment (MCI) compared to normal controls [7–10]. Dysbiosis indexed by alpha or beta diversities were also found in MCI and AD dementia patients [7–10]. However, specific alterations of the gut microbiome associated with AD dementia or MCI have been inconsistent among studies. Such inconsistent findings may in part result from the fact that dementia or cognitive impaired state itself could alter dietary patterns and lifestyles [11, 12] and therefore microbiome composition.

Cerebral Aβ deposition, the core pathology of AD, begins more than a decade earlier before cognitive symptoms appear [13]. Recent advances of AD neuroimaing biomarkers, particularly, amyloid positron emission tomography (PET) makes it possible to detect individuals in the preclinical stage of AD who have Aβ deposition in their brain but are still cognitively unimpaired [14, 15]. Early therapeutic intervention at the preclinical stage is getting more attention as evidence supporting the risk of clinical progression from the preclinical AD accumulates [13, 16].

To clarify the contribution of gut microbial alteration to cerebral Aβ pathology independently of the influence of dementia or cognitive impairment including dietary habit and lifestyle changes [11, 12], it is necessary to investigate preclinical AD patients. Yet, to date, few information is available for the gut microbiota in preclinical AD. Against this background, we aimed to identify gut microbial alterations associated with preclinical AD by comparing the gut microbiome in cognitively normal (CN) old adults with cerebral Aβ deposition (Aβ+ CN) and in those without cerebral Aβ deposition (Aβ− CN).

## Methods

### Participants

This study was conducted as part of the Korean Brain Aging Study for Early Diagnosis and Prediction of Alzheimer’s Disease (KBASE) [17], an ongoing prospective study launched in 2014. In the present study, we included 78 CN participants between 65 and 90 years of age. The CN subjects did not have dementia or MCI and had global Clinical Dementia Rating (CDR) scores of 0. The exclusion criteria were as follows: 1) presence of a major psychiatric illness; 2) significant neurological or medical conditions that could affect mental function; 3) current serious gastrointestinal (GI) disorders that could affect gut microbiome including GI tract cancers and inflammatory bowel diseases; 4) contraindications for an MRI scan (e.g., pacemaker or claustrophobia); 5) illiteracy; 6) the presence of significant visual/hearing difficulties and/or severe communication or behavioral problems that would make clinical examinations or brain scans difficult; 7) currently taking any antibiotics; 8) taking an investigational drug; and, 9) pregnant or breastfeeding. All participants underwent standardized clinical assessments performed by trained psychiatrists based on the KBASE clinical assessment protocol, which includes the Korean version of the Consortium to Establish a Registry for Alzheimer’s Disease (CERAD-K) Assessment Packet [17, 18]. This study was approved by the Institutional Review Board of Seoul National University Hospital in Seoul, Republic of Korea. The study protocol followed the recommendations of the current version of the Declaration of Helsinki. All participants gave written informed consent.

### Measurement of cerebral amyloid deposition

We used a 3.0T Biograph mMR (PET-MR) scanner (Siemens Healthcare, Erlangen, Germany) according to the manufacturer’s approved guidelines to acquire 3D [^11^C] Pittsburg compound B (PiB)–PET images, simultaneously with 3D T1-weighted MRI. We injected 555 MBq of ^11^C-PiB (range, 450–610 MBq) intravenously and obtained a 30-min emission scan 40 minutes after infusion. The PiB-PET data collected in list mode were processed for routine corrections such as uniformity, UTE-based attenuation, and decay corrections. Images were reconstructed into a 256 × 256 image matrix using iterative methods (six iterations with 21 subsets). For each participant, inverse transformation parameters were obtained from SPM12 DARTEL segmentation procedure using individual T1 images obtained on the same day as the PiB-PET and MNI template. These parameters were applied to the automatic anatomic labeling (AAL) atlas to acquire AAL atlas in native space for each participant, which were then used to extract PiB retention levels. Retention in cerebellum was separately extracted using a spatially unbiased atlas template of the cerebellum and brainstem (SUIT) to improve intensity normalization process [19]. Cerebellar gray matter was used as the reference region for quantitative normalization of cerebral PiB uptake. Using an AAL algorithm [20] and a region-combining method [21], the regions of interest (ROIs) were determined. The mean PiB retention levels in the frontal, lateral parietal, precuneus–posterior cingulate, and lateral temporal regions were divided by the mean cerebellar uptake value to yield a standardized uptake value ratio (SUVR) value for each ROI. Subjects were defined as Aβ+ CN if the SUVR value for at least one of the four ROIs was >1.4, and as Aβ− CN if the SUVR values of all four ROIs were ≤1.4 [21].

### Microbial sample collection and preparation

Stool samples were collected from the enrolled participants with guidance of experts. In details, every participant was guided to wear sterile gloves and collect stool by using sterile collection paper (JeongHyun MED, Goyang-si, Republic of Korea). Then, stools were stored in DNA/RNA shield buffer (Zymo Research, Irvine, CA, USA) for protecting from DNA a degradation at –80°C until used. Metagenomic DNA extractions were performed on 500 mg of feces per sample using a FastDNA® SPIN Kit for Soil (MP Biomedicals, Solon, CA, USA) according to manufacturer’s recommendations. DNA purity and quantity were estimated using a NanoDrop One Spectrophotometer (Thermo Scientific, Wilmington, DE, USA).

### 16S rRNA gene PCR amplification, sequencing, and processing

The extracted bacterial genomic DNA was used as a template for PCR amplification of the V3–V4 region of the bacterial 16S rRNA gene. Amplification was performed according to the Illumina 16S rRNA Sequencing Library Preparation guide (Illumina, San Diego, CA, USA) using the following primers with an added adapter overhang sequence [22]: forward, 5′- TCGTCGGCAGCGTCAGATGTGTATAAGAGACAGCCTACGGGNGGCWGCAG -3′; reverse, 5′-GTCTCGTGGGCTCGGAGATGTGTATAAGAGACAGGACTACHVGGGTATCTAATCC-3′. PCRs were performed in a 25-μL reaction volume containing 2 μL of genomic DNA (10 ng/μL), 0.5 μL of each primer (10 μM), 12.5 μL of 2× KAPA HiFi HotStart ReadyMix (Kapa Biosystems, Wilmington, MA, USA), and 9.5 μL of distilled water. PCR conditions were as follows: initial denaturation at 95°C for 3 min; 25 cycles consisting of denaturation at 95°C for 30 s, annealing at 55°C for 30 s, and extension at 72°C for 30 s; and a final extension at 72°C for 5 min. The PCR products were purified with AMPure XP Beads (Beckman Coulter, Brea, CA, USA) according to the manufacturer’s protocol. The attachment of dual-index sequences and Illumina adapters was conducted using 5 μL of the PCR product, 5 μL of Illumina Nextera XT Index Primer 1 (N7xx), 5 μL of Nextera XT Index Primer 2 (S5xx), 25 μL of 2× KAPA HiFi HotStart Ready Mixq, and 10 μL of nuclease-free water. Thermocycling was performed as follows: 95°C for 3 min; 8 cycles of 95°C for 30 s, 55°C for 30 s, and 72°C for 30 s; and a final extension at 72°C for 5 min. PCR products were purified with AMPure XP beads, and the quality control for the 16S rRNA libraries was performed using the Agilent Technologies 2100 Bioanalyzer (Agilent, Santa Clara, CA, USA). Libraries were normalized and pooled for sequencing on the MiSeq platform (Illumina) by 2×300 bp-paired end sequencing following standard Illumina sequencing protocols. The quality of the raw sequence reads was analyzed using FastQC [23]. Illumina adapter sequences of the paired-end reads were removed using Cutadapt version 2.2 [24]. Then, the trimmed sequences were processed using QIIME2 version 2019.7 [25]. Briefly, the reads were assigned to each sample according to a unique index; pairs of reads from the original DNA fragments were merged using an import tool in QIIME2 [25]. Quality control and trimming were performed to yield sequences with lengths of 230 and 220 bp for the forward and reverse reads, respectively. To remove low-quality bases at the end of the reads, the DADA2 software package [26] wrapped in QIIME2 was applied. To remove chimeras from the FASTQ files, a consensus method implemented in DADA2 was used. Taxonomic annotation was performed by mapping the training reference set with primers (forward, 5′-CCTACGGGNGGCWGCAG-3′; reverse, 5′-GACTACHVGGGTATCTAATCC-3′) and extracting the V3–V4 region using GreenGenes version 13_8 [27]. We performed a normalization of read counts. Coverage was calculated by feature counts/all sequence counts for each sample. As a result, the median of coverage of Aβ− CN was 43.592, while that of Aβ+ CN was 43.221. The coverages of each group were not also significantly different (P = 0.62, Wilcoxon-Mann-Whitney test).

### Statistical analysis

Demographic and clinical variables were compared using independent t-tests for continuous variables and Chi-square tests for categorical variables. Differences in genera between the Aβ+ and Aβ− CN groups were analyzed by a generalized linear model (GLM) using the R package “glmmTMB”. In this model, a zero-inflated negative binomial distribution was employed, and sequence counts of each taxon were treated as a dependent variable. A group factor (Aβ+ and Aβ− CN groups) was added to the model as an independent variable, with age, gender, body mass index (BMI), and apolipoprotein E ε4 (APOE4) positivity as covariates. Only taxa with a prevalence >0.1 were included in the analyses [28], and the natural log of total sequence counts for each participant was used as the offset. P-values were adjusted using the false-discovery-rate method and q-values <0.05 were considered significant. In addition, we constructed a multivariate logistic regression model based on the genera that differed significantly between Aβ+ and Aβ− CN in the GLM analyses. In this model, Aβ positivity was entered as a dependent variable, and the sequence counts of each genus were coded into dichotomous factors (i.e., 1 (presence) and 0 (absence)) and entered to the model together with the abovementioned covariates. The model was tested for a goodness of fit using Hosmer-Lemeshow Test and its performance was measured using the area under the curve (AUC) of receiver operating characteristic (ROC) curve. We also compared the AUC of the model with that of the model with only covariates (i.e., age, sex, BMI, APOE4) using the Delong method.

For exploration, we compared microbial pathways of Kyoto Encyclopedia of Genes and Genomes (KEGG) orthologs between the Aβ+ and Aβ− groups. Phylogenetic Investigation of Communities by Reconstruction of Unobserved States 1 (PICRUSt) based on operational taxonomic units (OTUs) was used for the comparison (version 1.1.4) [29]. P-values <0.05 were considered significant.

Alpha diversities measured as evenness, observed species, Shannon Index, and Faith’s phylogenetic diversity were compared between the Aβ+ and Aβ− CN groups using the Kruskal–Wallis test. Beta diversity was compared by principal coordinate analysis using Bray–Curtis distances, weighted and unweighted UniFrac metrics, and Aitchison distances between the two groups. Beta diversity between the groups was evaluated using permutational multivariate analysis of variance (PERMANOVA) with 999 permutations. P-values <0.05 were considered significant. All statistical analysis, unless otherwise mentioned, was performed using the R Statistical Software (version 4.0.2; R Foundation for Statistical Computing, Vienna, Austria).

## Results

### Participant characteristics

As shown in Table 1, there were no significant differences in age, gender, education, APOE4 positivity, BMI, and the presence of DM and HTN between the Aβ+ and Aβ− groups. As expected, there was a significant difference in global Aβ retention between the two groups. Using 16S rRNA sequencing, we identified a total of 227 fecal microbiome genera and 333 species in the participants. Most abundant 5 genera were *Bacteriodes*, *Prevotella*, *Faecalibacterium*, *Unclassified Lachnospiraceae*, and *Coprococcus*. Among 333 species, 149 gut microbiome species were commonly found in all participants, while the numbers of observed gut microbiome species were 231 for the Aβ+ group and 312 for the Aβ− group.

**Table 1:** Participant characteristics.

	Aβ− CN	Aβ+ CN	p-value
	(N = 60)	(N = 18)	
Age, yr	72.9 ± 6.8	75.2 ± 7.1	0.229
Gender (Female)	29 (48.3%)	11 (61.1%)	0.424
Education, yr	12.07	10.78	0.342
APOE4 Positivity	13 (21.7%)	6 (33.3%)	0.345
Global Aβ deposition (SUVR)	1.075 ± 0.074	1.800 ± 0.293	<0.001
BMI	24.3 ± 2.8	24.0 ± 3.2	0.741
Hypertension	32 (53.3)	10 (55.6)	0.868
DM	13 (21.7)	4 (22.2)	1.000

Note. Data are presented as mean ± SD or N(%). Either independent t test or Fischer’s exact test was used for comparison between groups

Abbreviations. Aβ+ CN: cognitively normal participants with amyloid retention Aβ− CN: cognitively normal participants without amyloid retention; Aβ: amyloid beta; APOE4: apolipoprotein E ε4; SUVR: standardized uptake value ratio; BMI: body mass index, DM: diabetes mellitus

### Differences in gut microbial taxa and classification performances of gut microbial taxa

**Fig 1:**
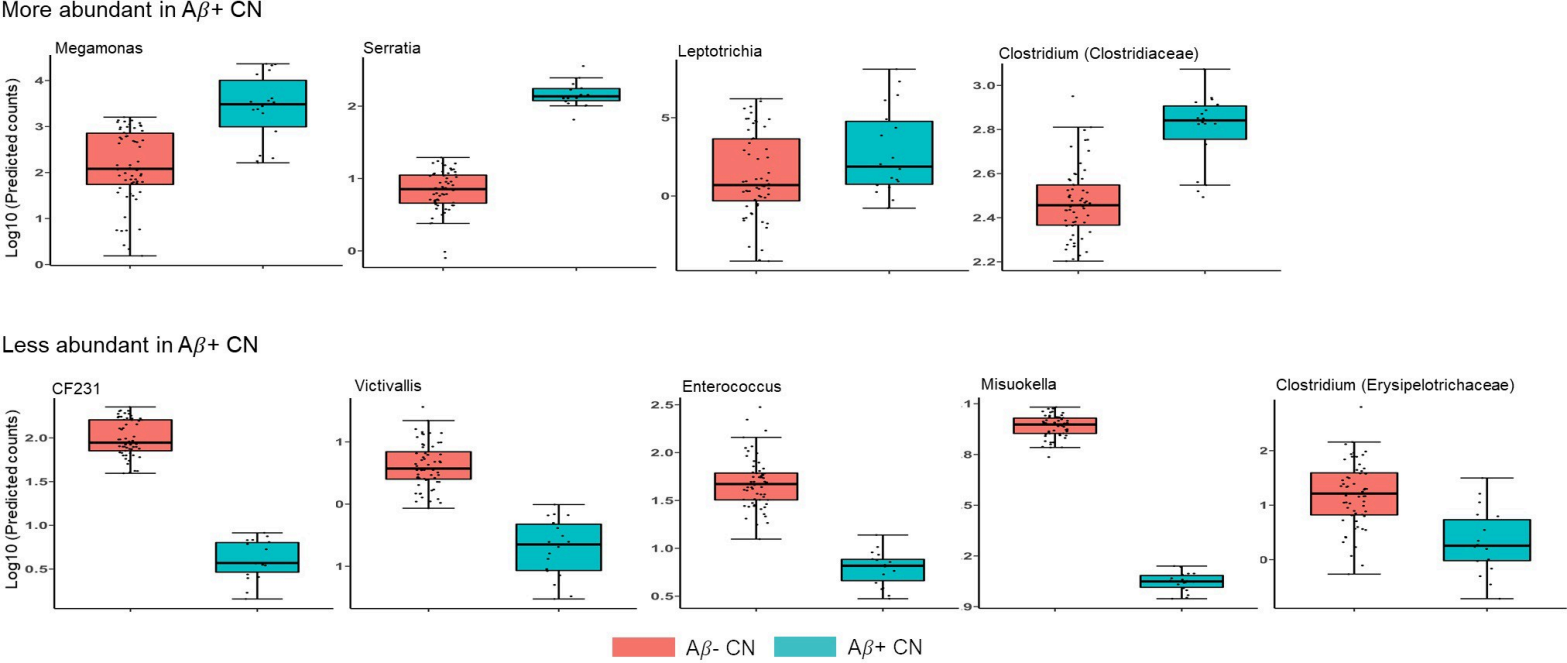
Log predicted counts of the genera associated with Aβ positivity. Prediction was made using GLM analysis adjusted for age, gender, BMI, APOE4 positivity. Only genera with q <0.05 are shown. Abbreviation. Aβ: amyloid beta; GLM: generalized linear model; Aβ+ CN: cognitively normal participants with amyloid retention Aβ− CN: cognitively normal participants without amyloid retention; APOE4: apolipoprotein E ε4; BMI: body mass index.

**Fig 2:**
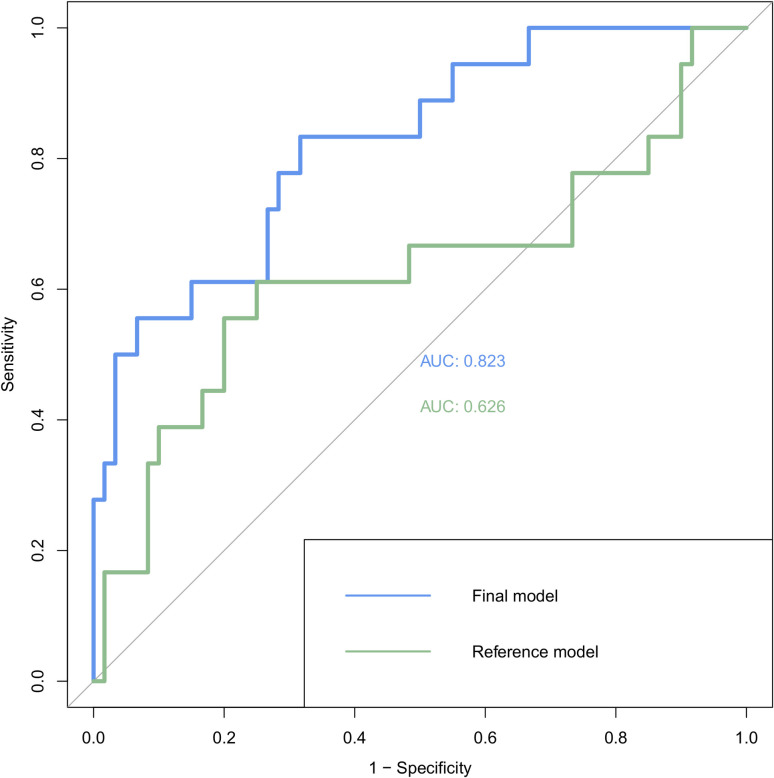
Receiver operating characteristic (ROC) curve analysis of the multivariate logistic model to detect preclinical AD using microbiome. The blue line represents the model with the presence or absence of nine genera revealed from the GLM analysis and covariates (i.e., age, sex, APOE4 positivity and BMI), while a green line represents the reference model including only the covariates. Abbreviation. AD: Alzheimer’s disease; APOE4: apolipoprotein E ε4; BMI: body mass index.

The GLM analysis revealed that the genera *Megamonas*, *Serratia*, *Leptotrichia* and *Clostridium* (family *Clostridiaceae*) were increased in the Aβ+ CN group compared to the Aβ− CN group, whereas the genera *CF231*, *Victivallis*, *Enterococcus*, *Mitsuokella* and *Clostridium* (family *Erysipelotrichaceae*) were decreased in Aβ+ CN (Table 2 and Fig 1). In the ROC analysis, the logistic regression model with variables representing the presence or absence of each of the nine genera effectively distinguish Aβ+ and Aβ− CN with the AUC value of 0.823 (95% CI 0.713–0.934) (Fig 2). The Hosmer-Lemeshow test indicated a good fit (p = 0.727). Moreover, when compared with the reference model, the model with the microbial genera was superior in distinguishing Aβ+ from Aβ− (AUC 0.823 vs 0.626, p = 0.003) (Fig 2).

**Table 2:** GLM analysis results of taxonomic differences in microbiome between Aβ+ CN participants and Aβ− CN participants.

Bacterial genus	Aβ− CN (%)	Aβ+ CN (%)	B value	SE	Z	p-value	q-value
*Megamonas*	0.804	0.441	3.399	0.437	7.787	<0.001	<0.001
*CF231*	1.39E-01	5.13E-03	-3.237	0.626	-5.171	<0.001	<0.001
*Victivallis*	8.63E-03	4.20E-04	-3.447	0.913	-3.777	<0.001	0.004
*Serratia*	0.014	0.107	3.044	0.835	3.644	<0.001	0.005
*Leptotrichia*	0.003	0.019	5.862	1.859	3.152	0.002	0.024
*Clostridium (family Clostridiaceae)*	0.436	0.918	0.788	0.263	2.992	0.003	0.034
*Enterococcus*	0.075	0.011	-2.044	0.728	-2.807	0.005	0.042
*Mitsuokella*	1.51E-01	6.38E-03	-2.119	0.759	-2.790	0.005	0.042
*Clostridium (family Erysipelotrichaceae)*	5.67E-02	2.44E-03	-2.222	0.805	-2.760	0.006	0.043

Note. Results of the GLM analysis adjusted for age, gender, BMI, and APOE4 positivity with Aβ− CN as the reference along with relative abundances (%) for each group. Only genera with q-values <0.05 are shown.

Abbreviations. GLM: generalized linear model; Aβ+ CN: cognitively normal participants with amyloid retention; Aβ− CN: cognitively normal participants without amyloid retention; APOE4: apolipoprotein E ε4; BMI: body mass index.

### Comparison of predicted functional microbiome pathways

**Fig 3:**
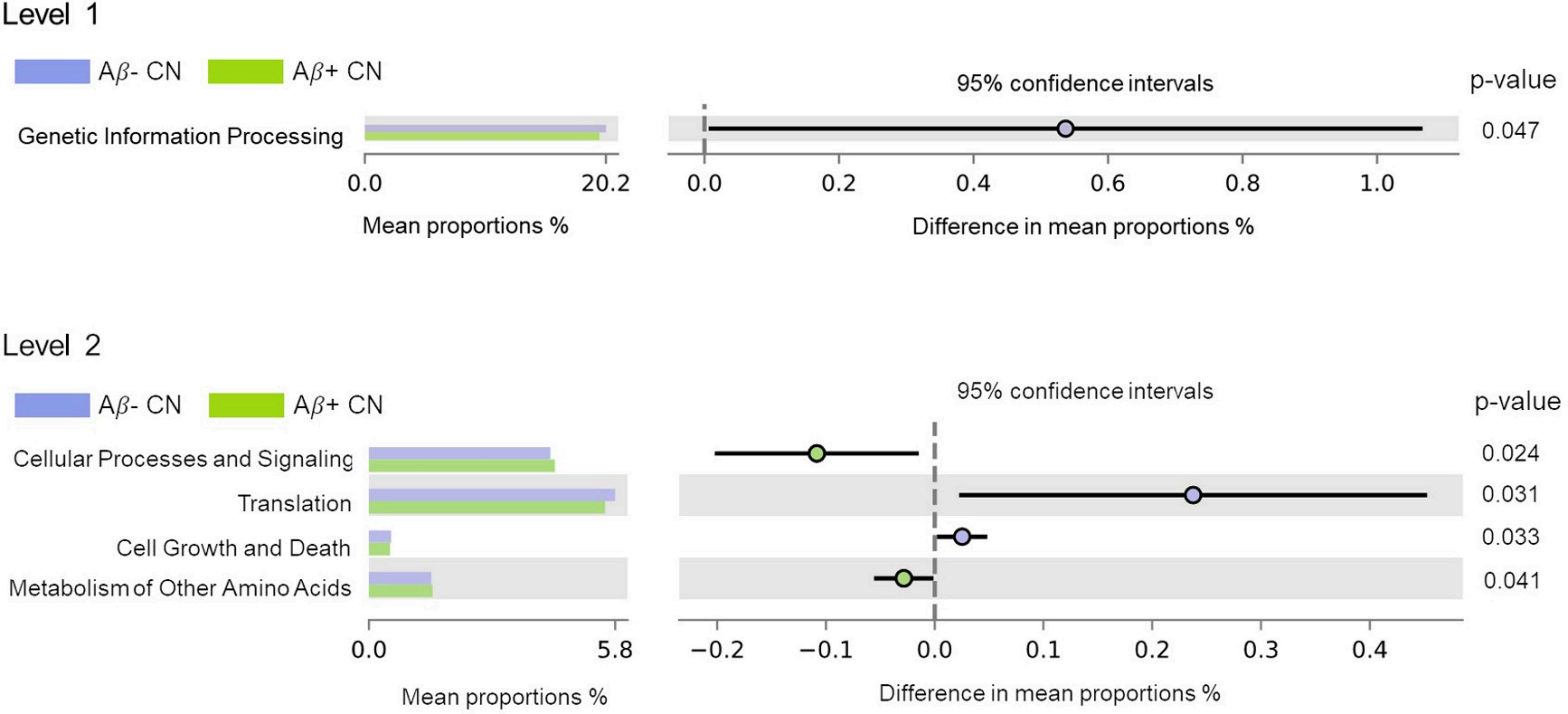
Relative differences in predicted gene contents in microbiome between Aβ− CN participants and Aβ+ CN participants. Abbreviations. Aβ+ CN: cognitively normal participants with amyloid retention Aβ− CN: cognitively normal participants without amyloid retention.

With regard to the KEGG functional pathways predicted using PICRUSt, a decrease in the pathway (i.e., gene contents) related to genetic information processing was found in the Aβ+ CN group compared to the Aβ− CN group in level 1 (Fig 3). In level 2, pathways related to translation and to cell growth and death were decreased in Aβ+ CN, whereas those related to cellular processes and signaling and to metabolism of other amino acids were increased compared to Aβ− CN (Fig 3 and S1 Table).

### Differences in diversity indices

There were no significant differences in any of the alpha diversity indices for fecal microbiota between Aβ+ and Aβ− (S1 Fig). Similarly, we found no significant beta diversity between Aβ+ and Aβ− (S2 Fig).

## Discussion

In our study, we found significant alterations in gut microbiome associated with Aβ positivity in CN elderly groups. Although many previous studies reported various gut microbial alterations in older adults with cognitive impairment such as AD dementia and MCI [7–9], little information is yet available for such alterations in cognitively unimpaired individuals with cerebral amyloid deposition, i.e., preclinical AD. We added evidence for gut microbial changes specifically associated with preclinical stage of AD process.

Four genera were increased, and five genera were decreased, in Aβ+ CN compared to Aβ− CN. Among the four genera that were increased in the Aβ+ CN group, i.e., *Megamonas*, *Serratia*, *Leptotrichia*, and *Clostridium (*family *Clostridiaceae)*, *Megamonas* was previously linked to Pre-DM [30], gestational DM [31], and obesity [32]. Given that both midlife obesity and DM are associated with increased risk of AD dementia or increased AD pathology [33, 34], *Megamonas* may contribute to cerebral Aβ deposition via its role in metabolic diseases. *Serratia* is known for occasional opportunistic infections [35], but its other roles in human health are largely unknown. *Leptotrichia* has been associated with periodontal disease, and its increase in the saliva microbiome was previously linked to AD [36]. The pro-inflammatory nature of this microorganism may contribute to AD not only through the oral cavity but also via the intestinal tract. The genus *Clostridium* (family *Clostridiaceae*) represents a large set of species comprising many pathogenic and commensal bacteria, including *Clostridium difficile* [37]. In accordance with our result, a decreased *Clostridium* level induced by probiotic treatment and exercise was associated with better cognitive performance in an AD mouse model [38]. In the present study, the most abundant species of genus *Clostridium* (family *Clostridiaceae*) was *Clostridium celatum*, whose level was also positively associated with amyloid positivity when we further analyzed the data. Although additional confirmatory studies are needed, the possible pathogenicity of *C*.*celatum* [39] and other known pathogenic *Clostridia* such as *C*.*difficile* may be related to harmful effects exerted by this genus on the brain.

Among the five genera that were decreased in the Aβ+ group, *i*.*e*., *CF231*, *Victivallis*, *Enterococcus*, *Mitsuokella* and *Clostridium (family Erysipelotrichaceae)*, *Enterococcus* is ubiquitous microorganism that produces lactic acid and has demonstrated probiotic potential [40]. The genus is also known to have anti-inflammatory properties [41] and to produce long chain fatty acids (LCFA), which could reduce obesity [42]. Thus, decreased *Enterococcus* may accelerate brain Aβ deposition through increased inflammation or altered lipid metabolism [34, 43]. In consistent with our results, a previous study reported reduced *Clostridium* (Family *Erysipelotrichaceae*) in AD dementia participants [10]. Nevertheless, *Clostridium ramosum*, the representative species of *Clostridium* (family *Erysipelotrichaceae*), was reportedly associated with obesity and increased energy absorption in a mouse model [44] and with DM in humans [45]. The genus *CF231* was also associated with overweight in Korean participants [46]. Given that the relation of body weight with AD is complex, as the timing and degree of obesity matter [47], further research on the association of AD with microbiome changes and metabolic diseases is needed to clarify the relationship. The inverse relationship of *Mitsuokella* and *Victivallis* with Aβ positivity is difficult to explain because their role in human health is largely unknown.

Considering all the potential links between the genera and pathophysiological changes mentioned above paragraphs, inflammatory or metabolic changes may mediate the relationship between the microbial changes we found and Aβ increase in preclinical AD. The mediation of inflammation was also suggested by previous studies on gut microbial changes in AD dementia and MCI patients [7, 48]. A study revealed altered pro- and anti- inflammatory microbiome in cognitively impaired Aβ+ participants [48]. Another study also demonstrated that gut microbiome associated with anti-inflammatory pathway was changed in AD dementia participants [7]. In regard of the mediation by metabolic changes, bacterial genera associated with DM or obesity were found to be altered in AD dementia participants [10]. However, as we did not directly examine the involvement of inflammation or metabolic changes related to microbial alterations, further studies are necessary to confirm the possibilities.

With the logistic regression model including the nine genera, we can effectively discriminate Aβ+ CN from Aβ−CN. The ROC analyses revealed fairly good AUC value. While this is generally line with previous reports that microbiome can distinguish amnestic MCI and AD from healthy controls [8, 9], our result further suggest that the microbiome may be helpful in detecting or screening preclinical or asymptomatic AD.

When we additionally analyzed functional aspects of the microbiome using PICRUSt, Aβ+ CN exhibited relatively low gene contents in pathways associated with genetic information processing such as translation or with cellular growth and death. These findings imply that cerebral amyloid retention may be associated with decreased vital genetic processes of gut microbiome. Similar to our results, a previous study reported decreased transcription, another step in genetic information processing, in AD dementia and MCI compared to healthy controls [9]. We also found that predicted gene contents related to cellular processes and signaling and to other amino acids metabolism were increased in Aβ+ CN. Other amino acid metabolism refers to the metabolism of non-proteinogenic amino acids such as beta alanine or D-amino acids. D-amino acids are key components of bacterial peptidoglycan [49], which is known to cause inflammatory processes in the human brain [50]. Bacterial peptidoglycan was also observed together with neuritic plaque in post-mortem brains of AD patients [51]. Furthermore, the beta-alanine level in feces was increased in the feces of patients with inflammatory bowel disease [52]. Thus, the inflammatory processes related to these metabolites may be the underlying link for the association found in this study. However, cautious interpretations of the results of the PICRUSt functional analysis of the microbiome are warranted, since unlike metagenomic sequencing, 16S rRNA sequencing can only infer functional profiles [29].

In this study, we also investigated the alpha and beta diversities of the gut microbiome. No differences were found in the alpha and beta diversities between Aβ+ and Aβ− CN participants. This result is not consistent with previous reports, which showed significant decrease of alpha diversity [8, 9] and significant beta diversity [7–9] in individuals with AD dementia or MCI. However, it should be noted that the current study targeted only Aβ+ CN (i.e., preclinical or asymptomatic AD), whereas all of the previous studies investigated cognitively impaired (i.e., AD dementia and MCI) patients. Dementia or a cognitively impaired state itself is commonly associated with poorer nutritional status and/or lower BMI [53] which could alter microbiome composition [54]. Moreover, most AD dementia patients and some MCI individuals take cholinesterase inhibitors, which can increase colonic transit time and affect bowel conditions related to the gut microbiome [55]. Therefore, the decreased alpha and beta diversity of gut microbiome reported in previous studies may not be directly linked to AD pathology but may, instead, reflect secondary phenomena caused by nutritional changes or medications common in cognitively impaired individuals.

A strength of our study is its role as the first to reveal gut microbiome alterations associated with preclinical AD independently of the influence of cognitive impairment. However, some limitations need to be mentioned. First, as this was a cross-sectional study, we cannot infer a causal relationship between gut microbial alterations and brain Aβ deposition. Further longitudinal research would be helpful to confirm the beneficial or detrimental effects of the microbiomes found in this study. Second, we did not assess the diet intake patterns of individual participants. Although CN individuals usually maintain their original dietary patterns [56, 57] and BMI was controlled as a covariate in the present study, individual differences in diet may confound the result. Additionally, detailed information about the past experience of antibiotics intake needed to be considered although we excluded individuals with current use of antibiotics. Third, other potential confounders such as physical activity, probiotic use, and alcohol consumption also need to be further controlled. Finally, the sample size was relatively small, which might have it difficult to reveal subtle differences between groups. Further studies with larger samples are needed to confirm the findings.

In conclusion, our findings suggest that specific alterations of gut bacterial taxa are related to preclinical AD and may be helpful for screening the preclinical AD.

## Supporting information

S1 Fig Comparison of α-diversities of the fecal microbiome between Aβ− CN participants and Aβ+ CN participants according to (A) evenness (B) observed species (C) Shannon index and (D) phylogenic diversity (PD). Abbreviations. Aβ+ CN: Cognitively normal participants with amyloid retention; Aβ− CN: Cognitively normal participants without amyloid retention; PD: phylogenetic diversity.
(TIF)

S2 Fig β-diversities of the fecal microbiome between Aβ− CN participants and Aβ+ CN participants based on (A) Bray-Curtis (B) unweighted UniFrac (C) weighted UniFrac and (D) Aitchison values. Abbreviations. Aβ+ CN: cognitively normal participants with amyloid retention; Aβ− CN: cognitively normal participants without amyloid retention.
(TIF)

S1 Table Comparison of relative abundances of predicted KEGG functional pathway analysis between Aβ+ CN participants and Aβ− CN participants. (PDF)
