# Supplementary figures and images for: Gut microbiome alterations in preclinical Alzheimer’s disease
Source: PLoS One. 2022 Nov 29;17(11):e0278276. doi: 10.1371/journal.pone.0278276 (PMC9707757; doi:10.1371/journal.pone.0278276)

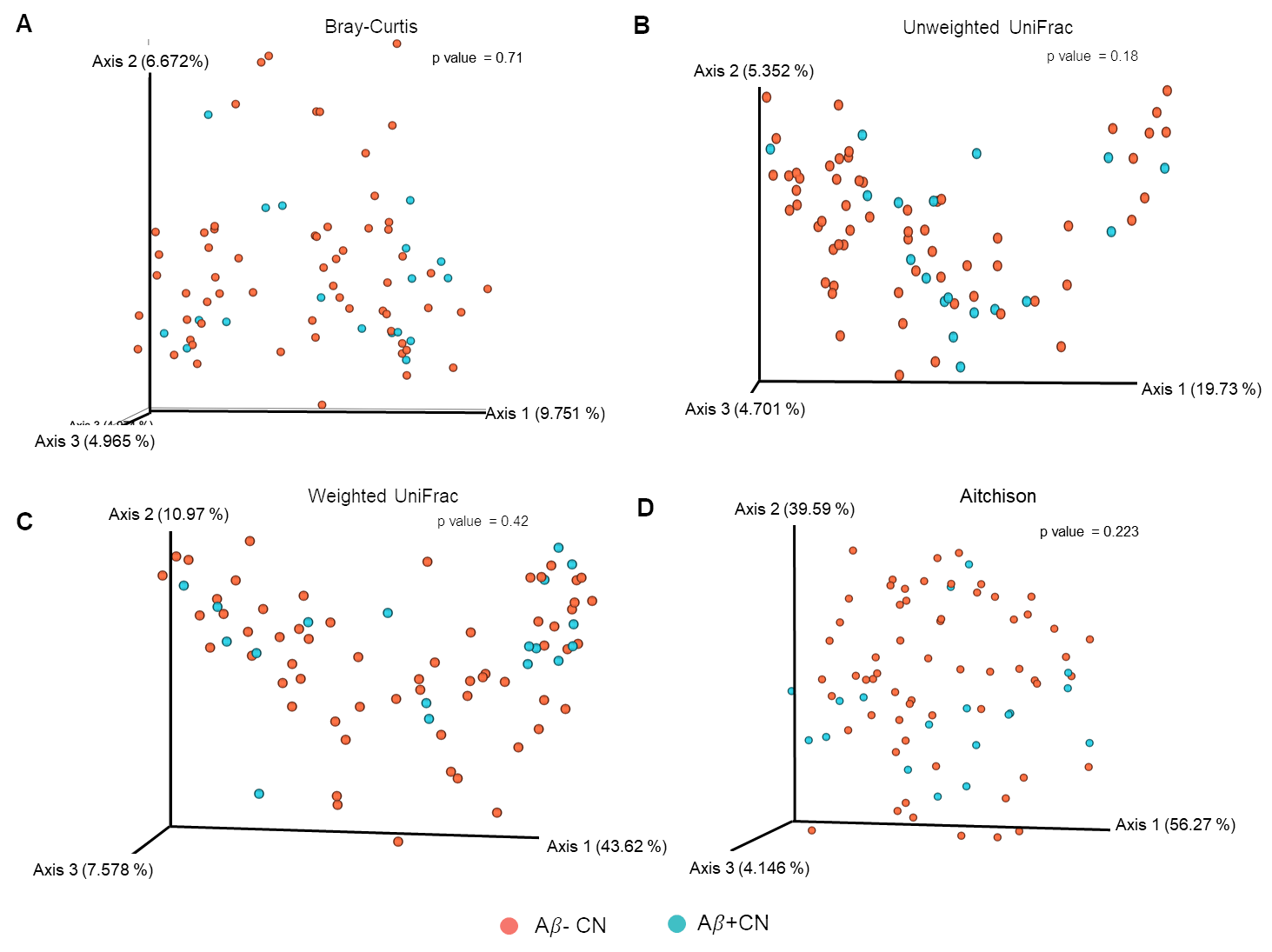

Supplement: S1 Fig — Comparison of α-diversities of the fecal microbiome between Aβ− CN participants and Aβ+ CN participants according to (A) evenness (B) observed species (C) Shannon index and (D) phylogenic diversity (PD). Abbreviations. Aβ+ CN: Cognitively normal participants with amyloid retention; Aβ− CN: Cognitively normal participants without amyloid retention; PD: phylogenetic diversity. (TIF) [file pone.0278276.s001.tif]

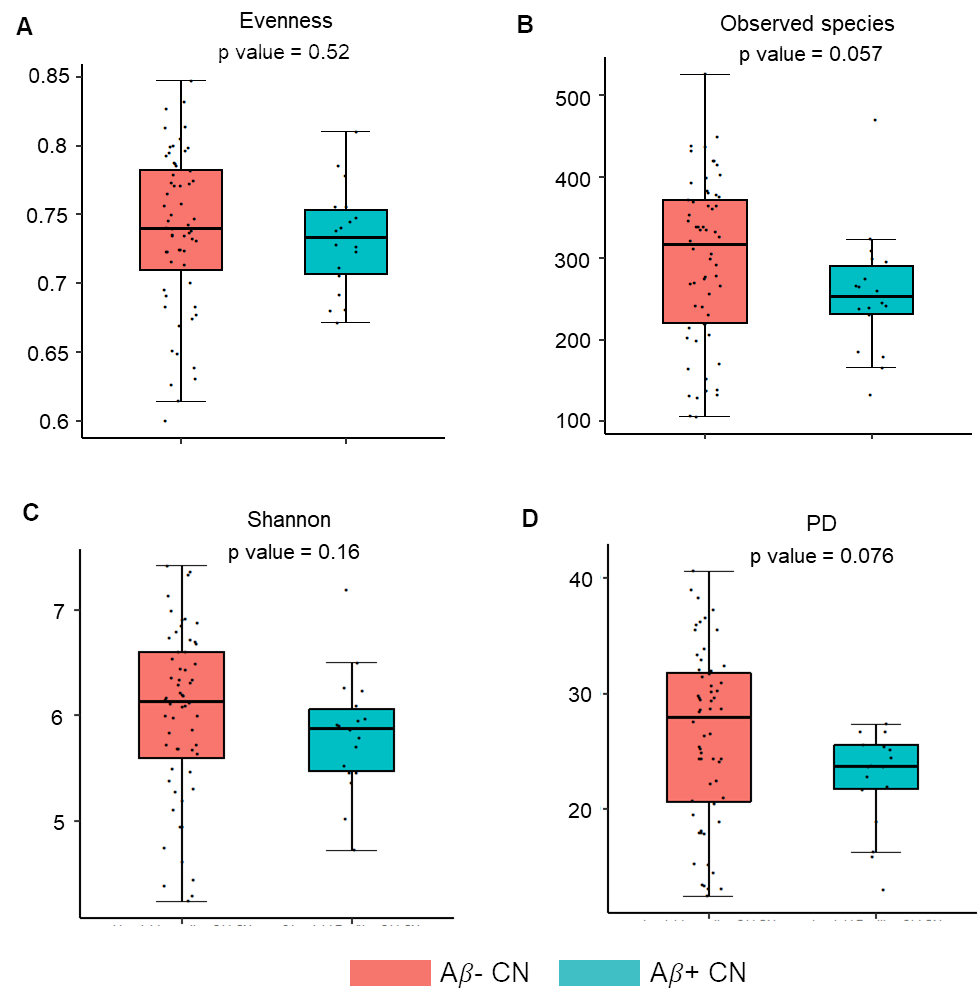

Supplement: S2 Fig — β-diversities of the fecal microbiome between Aβ− CN participants and Aβ+ CN participants based on (A) Bray-Curtis (B) unweighted UniFrac (C) weighted UniFrac and (D) Aitchison values. Abbreviations. Aβ+ CN: cognitively normal participants with amyloid retention; Aβ− CN: cognitively normal participants without amyloid retention. (TIF) [file pone.0278276.s002.tif]
