# Supplementary material for: Gut microbiome alterations in preclinical Alzheimer’s disease
Source: PLoS One. 2022 Nov 29;17(11):e0278276. doi: 10.1371/journal.pone.0278276 (PMC9707757; doi:10.1371/journal.pone.0278276)
Supplement: S1 Table — (PDF) [file pone.0278276.s003.pdf]

Supplementary Table S1. Comparison of relative abundances of predicted KEGG functional pathway analysis between groups

Level	Pathways	$A\beta-$ CN, relative abundance (%)	$A\beta+$ CN, relative abundance (%)	p-value
Level 1	Genetic information processing	20.23 (0.92)	19.69 (1.15)	0.047
Level 2	Cellular processes and signaling	4.25 (0.16)	4.36 (0.2)	0.024
	Translation	5.77 (0.37)	5.53 (0.47)	0.031
	Cell growth and death	0.52 (0.04)	0.50 (0.05)	0.033
	Metabolism of other amino acids	1.46 (0.05)	1.49 (0.05)	0.041

Note. Data are presented as mean (SD) %

Abbreviations. KEGG: Kyoto Encyclopedia of Genes and Genomes; Aβ+ CN: cognitively normal participants with amyloid retention Aβ− CN: cognitively normal participants without amyloid retention
